# Cytochrome *c* Oxidase on the Crossroads of Transcriptional Regulation and Bioenergetics

**DOI:** 10.3389/fphys.2019.00644

**Published:** 2019-05-24

**Authors:** Ivan Vladimirovich Chicherin, Erdem Dashinimaev, Mariia Baleva, Igor Krasheninnikov, Sergey Levitskii, Piotr Kamenski

**Affiliations:** ^1^Department of Molecular Biology, Faculty of Biology, Lomonosov Moscow State University, Moscow, Russia; ^2^Institute of Functional Genomics, Lomonosov Moscow State University, Moscow, Russia; ^3^Koltzov Institute of Developmental Biology, Russian Academy of Sciences, Moscow, Russia; ^4^Pirogov Russian National Research Medical University, Moscow, Russia

**Keywords:** mitochondria, cytochrome *c* oxidase, neurons, transcriptional control, supercomplexes, bioenergetics

## Abstract

Mitochondria are the organelles of eukaryotic cells responsible for the ATP production by means of the electron transfer chain (ETC). Its work is under strict genetic control providing the correct assembly of the enzyme complexes and the interface to adapt the energetic demands of the cell to the environment. These mechanisms are particularly developed in the cells with high energy consumption, like neurons and myocytes. This review summarizes several aspects of the involvement of the ETC complexes in the transcriptional control mechanisms of the neurons and other cells. Their influence on the differentiation of neurons is also discussed.

## Introduction

Mitochondria are essential organelles of eukaryotic cells. According to the currently accepted theory, they originated from the endosymbiosis of alphaproteobacteria with an ancient host cell (Roger et al., 2017). As the traces of the previous autonomy, mitochondria retain their own genome and gene expression system. However, the transition to the permanent organelles caused profound evolutionary changes (Roger et al., 2017). One of their main functions is the aerobic respiration, a complex process of pyruvate oxidation producing reducing equivalents further oxidized in the electron transfer chain (ETC) and production of ATP. This is why mitochondria are often assigned as the “powerhouses” of the cell. However, the involvement of the organelles in the life of the cell goes widely beyond this function and they participate in dozens of metabolic, biosynthetic and regulatory pathways.

Mitochondria form the reticulum network of the interconnected tubules with a complex membrane structure, comprising the inner and outer membranes divided by the intermembrane space. The inner membrane is heavily convoluted forming cristae, maintained by the ATP synthase dimers at their sharp edges (Plecitá-Hlavatá and Ježek, 2016). The respiratory function of mitochondria is provided by the ETC resides in the inner mitochondrial membrane. It consists of four enzyme complexes (I-IV) which catalyze electron transfer from NADH and FADH_2_ to molecular oxygen and pump the H^+^ ions in the intermembrane space generating the chemi-osmotic gradient across the membrane which is dissipated by the ATP synthase (often assigned as complex V) producing ATP.

Cell kind-specific variations in mitochondrial content and activity demonstrated remarkable differences in multiple tissues of different mammals (El-Merhie et al., 2017), suggesting the precise regulation of mitochondrial gene expression. According to the paradigm, these regulation mechanisms evolved to fine-tune energy production of the cells to fit their physiological states and the environmental requirements. This review summarizes several aspects of the involvement of the cytochrome *c* oxidase (COX or complex IV) and other complexes in the transcriptional control mechanisms of the cell. Particular interest is paid to the complex IV because it is best studied in this aspect and illustrates the principles, which may be applied to other complexes. The mechanisms and pathways we discuss here are highly developed in neurons, therefore particular attention is paid to these cells. Moreover, the data available for neurons demonstrates that mitochondria are responsible for the differentiation and identity of the cell, which does not challenge the existing paradigm, but completes it.

## COX is Involved in the Regulation of the ETC

COX accepts electrons from cytochrome *c* and transfers them to molecular oxygen, meanwhile pumping two H^+^ ions into the intermembrane space. According to recent findings, mammalian COX consists of 14 protein subunits (Zong et al., 2018). To avoid the discrepancies, all subunits will be named further according to the recently suggested nomenclature (Pitceathly and Taanman, 2018). The three largest subunits of the enzyme (I, II, and III) are encoded by mitochondrial DNA. They coordinate prosthetic metal groups and heme redox centers forming the catalytic site (Sinkler et al., 2017). All other subunits are encoded in the nucleus with the corresponding genes localized on several different chromosomes. This complicated genetic situation taken together with the critical function of the COX suggests a strict control of its assembly process on multiple levels coupled with the tight coordination of gene expression between two genomes. These regulating mechanisms providing stoichiometric assembly of all 14 subunits in a functional holoenzyme were actively probed in recent years; however, we are still far from the detailed understanding.

One of the key features in the regulation of the COX in mammals is the existence of alternative isoforms. Thus the subunits VIa, VIIa, and VIII have liver-specific isoforms ubiquitously expressed and muscle-specific isoforms expressed in heart and skeletal muscles (reviewed in Sinkler et al., 2017). All of them are small proteins encoded by separate nuclear genes, which arose by the gene duplication events during evolution. The liver-specific isoforms are substituted with the muscle-specific ones during the development of muscle cells (Sinkler et al., 2017). In addition to that, specific isoform of subunit IV is found in lungs, and the one of subunit VIb in testes (Hüttemann et al., 2003).

Complex IV is unique from this point of view, because no alternative isoforms have been reported for any other complex of the ETC. This fact suggests the additional level of controlling the COX activity due to its particular importance. One of the hypotheses explaining that is based on the calculations of the free energy released in enzymatic reactions of different complexes. In case of COX, this parameter is significantly higher than for complexes I and III, making the reaction irreversible and therefore deserving additional control mechanism (Sinkler et al., 2017). The role of the heart and muscle-specific subunits is to fit the activity of CIV in the cells to the high energy demands dictated by their functions. The molecular basis of this mechanism remains mysterious due the lack of the functional studies. Localized on the periphery of the complex, the small tissue-specific COX subunits VIa, VIIa and VIII may play the key role in the transition between dimeric and monomeric forms of the enzyme. Thus, the 14-subunit monomer contains exclusively VIIa-L, VIa-L, and VIII-L, while the alternative VIIa-H, VIa-H and VIII-H subunits are found in the dimer (Zong et al., 2018). Similar conclusions were made from the earlier studies: the complex IV dimer seemed to be favored by the concomitant presence of VIIa-H and VIa-H isoforms (Cogliati et al., 2016). Notably, the dimer-forming variants are found in mammalian tissues with highly active mitochondria (like heart and muscles), while the monomeric ones are expressed in tissues that generally contain fewer mitochondria (like brain, liver, and kidney) (Lobo-Jarne et al., 2018). It is not currently clear what impact do these stoichiometric transitions of CIV have on the efficiency of respiration and functions of the ETC, since the activity of the organelles does not correlate with their content in the cell. Interestingly, respirasomes, the assemblies containing CI+(CIII)_2_+CIV, are almost undetectable in most mammalian tissues expressing liver-specific COX isoforms (Cogliati et al., 2016), but it is unlikely to be connected with the monomeric state of CIV in these tissues. Taking into account the high energy consumption of neurons, it is surprising that no neuron-specific isoforms have been described so far. Moreover, the liver variants of the alternative subunits are expressed in these cells. This seems counterintuitive, because they are known to shift the topology of the respiration chain to the free complexes instead of forming respirasomes and increase the ROS production. One would expect that the ETC in neurons should be rather organized as in the heart and muscles, with highly developed supercomplex structure, but it does not seem to be the case (Lobo-Jarne et al., 2018). However, there is no solid proof that respirasomes make the energy production more efficient, which can explain their absence in neurons.

Remarkable differences in the organization of the respiratory chain are found in the cells of the nervous system. The brain functioning requires tight cooperation between neurons and astrocytes. While the neurons are the key functional units of mammalian brain, the astrocytes play rather supportive role by regulating the redox status and protecting the neurons and themselves from reactive oxygen species (ROS) due to their high content of antioxidants (Dringen et al., 1999). Neurons produce ATP by oxidative phosphorylation, whereas the energy supply of astrocytes rely on glycolysis (Almeida et al., 2004). These functional differences are reflected in the structural organization of the ETC. Thus, the astrocytes have complex I mostly in free form, but in neurons it is rather involved in the interactions with other enzymes and forms the supercomplexes (Lopez-Fabuel et al., 2016). This in turn leads to increased ROS production and less efficient respiration in astrocytes. The described situation provides an excellent example illustrating the interconnections between the organization of the respiratory chain, bioenergetics and physiology of different kinds of nervous cells.

It is traditionally considered that the identity of the cell governs the mitochondrial function. This point of view suggests that cell proliferation is a primary event, which adjusts the mitochondrial respiration and supercomplex assembly (secondary events) to fit the new energetic requirements of the cell and manage the ROS production. However, regarding the investigations in the area, the paradigm is questioned furthermore, and the opposite scenario is discussed (Parker et al., 2009). It is well known that human embryonic stem cells (hESC) sustain in low oxygen conditions. It has been demonstrated experimentally that spontaneous differentiation is significantly reduced in the hESC cultures in these conditions (Ezashi et al., 2005). At the same time, the undifferentiated hESC produce ATP mainly by glycolysis, and their mitochondria are low in number and undeveloped, with few cristae. As the cells differentiate, they switch to oxidative phosphorylation and their mitochondrial content and activity are increased (Cho et al., 2006). Regarding these data, it is tempting to assume that it is possible to affect the cell fate by influencing mitochondria. This is exactly the case demonstrated by a group of researchers who blocked the differentiation of neurons by the treatment with antimycin A, a specific inhibitor of complex III (Pereira et al., 2013). Upon such treatment the embryonic stem cells failed to differentiate into dopaminergic neurons, maintained low levels of neuronal differentiation markers and high levels of pluripotency markers. Interestingly, one of the possible explanations of the phenomenon is the increased ROS production observed in the antimycin A treated cells. This explanation seems logical in view of the previous data, and we can suggest that it happens because of poor involvement of complex I in the supercomplexes upon hypoxic conditions. To summarize, the development of mitochondria, their functional activity, ROS production and the composition of the respiratory chain supercomplexes are closely connected and critical for neuronal differentiation.

## COX Genes are Involved in Transcriptional Networks in Neurons

The brain is one of the highest energy-demanding organs of the body in mammals. It is composed of a heterogeneous population of neurons with different physiological and morphological characteristics. The neurons have multiple energy consuming activities, such as the synthesis of proteins and other molecules, turnover of transmitters and receptors, active anterograde and retrograde transport of proteins and organelles, and active transport of ions against their concentration and electrical gradients (Wong-Riley, 2012). The supply of energy in these cells comes almost entirely from oxidative phosphorylation, making them heavily dependent on mitochondrial function. It is not surprising that these cells developed sophisticated mechanisms controlling the expression of the corresponding genes. Neurons represent a brilliant example of the network regulated by transcriptional control mechanisms, providing the interconnection between the energy turnover, metabolism and functions of these cells.

The key players that certainly deserve discussing here are nuclear respiratory factors 1 and 2 (NRF-1 and NRF-2) and the mitochondrial transcription factor A (Tfam). Tfam controls transcription of mitochondrial DNA, and NRF-1 and NRF-2 are the primary transcription factors of nuclear DNA genes encoding mitochondrial proteins, including Tfam (Chen et al., 2009). The role of these and other transcriptional factors in neurons has been actively investigated in recent decades.

NRF-1 regulates transcription of ten nuclear-encoded COX subunits in murine neurons. The data of multiple *in silico*, genetic and biochemical experiments documented its binding sites, conserved among mice, rats and humans, on the corresponding promoters (Dhar et al., 2008). In addition to the genes encoding mitochondrial proteins, NRF-1 controls the expression of several genes crucial for neurons, thus mediating the tight coupling of neuronal activity, energy generation, and energy consumption at the molecular level. Among the known targets regulated by NRF-1 in neurons are Na^+^/K^+^-ATPase (Johar et al., 2012), the subunits 1 and 2b of glutamatergic *N*-methyl-d-aspartate (NMDA) receptor (Dhar and Wong-Riley, 2009), neuronal isoform of NO synthase (Dhar et al., 2009), the β1 subunit of γ-aminobutyric acid (GABA) subtype A receptor (Li et al., 2018). Na^+^/K^+^-ATPase is the main energy consuming enzyme capable for maintaining the depolarization/repolarization cycles by generating Na^+^ and K^+^ gradients across the neuronal plasma membrane (Johar et al., 2012). NMDA receptor is involved in the synaptic transmission of excitatory signals in the central nervous system and therefore plays an important role in neuronal plasticity, learning, and memory (Dhar and Wong-Riley, 2009). Nitric oxide synthase is the enzyme responsible for production of nitric oxide, a signaling molecule acting through the cGMP pathway (Dhar et al., 2009). The GABA-A receptor participates in inhibitory neurotransmission in the central nervous system by forming the Cl^–^ ion channels (Li et al., 2018). In all described cases the regulation mechanism remains the same as for the COX genes: NRF-1 recognizes the corresponding promoters and activates transcription. Its binding sites are well conserved in different mammals. NRF-1 represents a solid proof that that synaptic neuronal transmission and energy metabolism are tightly coupled at the molecular level.

Similar experimental approaches (computer sequence analysis, chromatin immunoprecipitation, electrophoretic mobility shift assays, etc.) were used to document NRF-2 is another master-regulator of ten nuclear-encoded COX subunits in rat neurons (Ongwijitwat and Wong-Riley, 2005). It also acts on transcriptional level. Besides the COX subunits, NRF-2 affects the mRNAs of other nuclear-encoded mitochondrial proteins, like TFAM, TOM20, SURF1, and VDAC1 and provides the link between the activity of gene expression and the energy demands of the cells (Ongwijitwat et al., 2006). As in case of NRF-1, NRF-2 has the additional regulation targets among the genes encoding the crucial neuronal proteins, like NMDA receptor (Priya et al., 2013a) and brain-derived neurotrophic factor (BDNF) in rat visual cortex (Nair and Wong-Riley, 2016). Curiously, both NRF-1 and NRF-2 regulate the expression of the same subunits 1 and 2b of NMDA receptor. BDNF is regarded as the most widely expressed, active, and well characterized neurotrophin in developing and adult mammalian central nervous system. The gene is differentially expressed in different brain regions as well as in peripheral tissues (Nair and Wong-Riley, 2016).

It is worth noting that besides the nuclear encoded COX subunits, both NRF-1 and NRF-2 certainly affect the mitochondrial COX genes (*COX1, COX2*, and *COX3*), although their mode of action is indirect in this case. It is mediated by regulating the crucial proteins involved in mitochondrial gene expression and metabolism. The list includes the factors of mitochondrial DNA replication (Tfam, mtSSB and hSUV3 DNA helicase), transcription (TFB1M and TFB2M), translation (MTIF2) and protein import and assembly machineries (TOM20, SURF1, and COX17) (reviewed in Chen et al., 2009). Certainly, other respiration chain complexes are also controlled by these broad-range transcription factors. Thus, the expression of the genes encoding the subunits of complex II (all of them are nuclear-encoded) is regulated by the direct mechanism (Piantadosi and Suliman, 2008), and those of the complexes I and III are under indirect control (Chen et al., 2009).

Besides NRF-1 and NRF-2, promoters of a number of nucleus-encoded COX genes in neurons contain the binding sites for specificity proteins Sp1 and Sp4. Recently both Sp1 and Sp4 were identified as transcription factors of COX genes as well as for the genes encoding subunits of the receptors very similar to NRF-1 and NRF-2. Thus, Sp1 functionally regulates the 10 nucleus-encoded COX subunit genes directly and the three mitochondrial COX subunit genes indirectly by regulating mitochondrial transcription factors A and B (TFAM, TFB1M and TFB2M) in neurons (Dhar et al., 2013). This regulation is intimately associated with neuronal activity: silencing of Sp1 prevented the upregulation of all COX subunits by KCl, and over-expressing Sp1 rescued all COX subunits from being downregulated by tetrodotoxin, the specific inhibitor of Na^+^ channels (Dhar et al., 2013). However, the discussed pathways are documented only in neuroblastoma N2a cells, but in primary neurons Sp1 is functionally replaced with another factor, Sp4, specific for the neurons and testicular cells. Accordingly, the nuclear content of Sp4 is significantly higher in primary neurons than in neuroblastoma cells (Dhar et al., 2013). Sp4 was found to do practically the same job as Sp1 – bigenomic regulation of the transcription of all mitochondria- and nucleus-encoded genes of COX subunits in neurons (Johar et al., 2013). As in previous case, the nuclear-encoded subunits are regulated by direct interaction with the promoters. Mitochondrial subunits are controlled indirectly by managing the mitochondrial transcription (TFAM, TFB1M, and TFB2M) and the COX assembly factors (SURF-1) (Johar et al., 2013). Similar to NRF-1 and NRF-2, the expression of multiple genes encoding the receptor subunits are under transcriptional control of Sp4. These targets are the subunits Atp1a1, Atp1a3 and Atp1b1 of Na^+^/K^+^-ATPase (Johar et al., 2014), the subunits GluN1, GluN2A, and GluN2B of NMDA receptor (Priya et al., 2013b), the subunit GluA2 of AMPA receptor (Priya et al., 2014) and several subunits of GABA-A receptors (Nair et al., 2016).

To summarize, the neurons possess the deeply developed system of transcriptional control by the broad-range specificity factors responsible for the expression of the set of their essential genes. These genes encode two groups of proteins: the neuron-specific and mitochondrial ones. The first group contains the signature proteins that define the identity of the neural cells (receptors, ion channels and enzymes) due to their functional role in membrane polarization, neurotransmission and recognition of the signal molecules. The second group contains the proteins involved in different aspects of mitochondrial gene expression and metabolism. The complexes of the ETC, being a part of the second group, occupy particular position in this network because they are encoded bigenomically, which requires additional efforts providing their stoichiometric assembly. This neighborhood makes the researchers suggest that highly developed mitochondria are one of the key characteristics of the neurons making large impact in defining of the identity of these cells. At this point, the COX, the best studied example of multiple-leveled transcriptional control in neurons, is even suggested as the critical metabolic markers of neuronal activity (Wong-Riley, 1989).

## Concluding Remarks and Future Perspectives

In this review we analyzed the data available about the regulation of the genes encoding the ETC complexes in neurons. The COX genes are deeply involved in the transcriptional networks of the cells and regulated by the broad-range transcription factors, responsible for the signature neuronal enzymes and receptors. The COX activity can serve as the biomarker of neurons and most likely is crucial for their differentiation.

In future the regulatory networks discussed here can be extended and include the components of mitochondrial translation, protein import and assembly machineries. The data about that is rather limited. MITRAC complex, containing the newly synthesized COX subunits along with the components of TIM23 translocase is known to link these processes together (Mick et al., 2012), however we do not know if these interactions are involved in the assembly of other complexes. The influence of the activity of the ETC complexes on the proliferation of different kinds of cells is certainly underinvestigated. These questions remain to be solved in future.

## Author Contributions

IC built the plot of the article and wrote the abstract and the Introduction. MB and SL wrote the section “COX Is Involved in the Regulation of the ETC.” ED wrote the section “COX Genes Are Involved in Transcriptional Networks in Neurons.” IK prepared the text for publication. PK did the language editing, proofing and did the critical reading and revision of the manuscript.

## Conflict of Interest Statement

The authors declare that the research was conducted in the absence of any commercial or financial relationships that could be construed as a potential conflict of interest.
